# Do we have (in)compatibilist intuitions? Surveying experimental research

**DOI:** 10.3389/fpsyg.2024.1369399

**Published:** 2024-04-22

**Authors:** Kiichi Inarimori, Souichiro Honma, Kengo Miyazono

**Affiliations:** ^1^Laboratory of Philosophy and Ethics, Department of Philosophy and Religious Studies, Graduate School of Humanities and Human Sciences, Hokkaido University, Sapporo, Japan; ^2^Center for Human Nature, Artificial Intelligence, and Neuroscience, Hokkaido University, Sapporo, Japan

**Keywords:** free will, experimental philosophy, moral responsibility, intuition, compatibilism, incompatibilism

## Abstract

This article critically examines the experimental philosophy of free will, particularly the interplay between ordinary individuals’ compatibilist and incompatibilist intuitions. It explores key insights from research studies that propose “natural compatibilism” and “natural incompatibilism”. These studies reveal a complex landscape of folk intuitions, where participants appear to exhibit both types of intuitions. Here, we examine error theories, which purport to explain the coexistence of apparently contradictory intuitions: the Affective Performance Error hypothesis, the “Free Will No Matter What” hypothesis, the Bypassing hypothesis, and the Intrusion hypothesis, and the article explores the cognitive errors that could shape individuals’ inconsistent perceptions of free will. We then explore three possibilities regarding folk intuitions: most individuals may hold either compatibilist or incompatibilist intuitions, both simultaneously, or neither. Our aim is to deepen the understanding of the complex dynamics of intuitions about free will, and we close with suggestions for future studies in experimental philosophy.

## 1 Introduction

Imagine that I suddenly struck you. You would probably be taken aback, exclaim something like “What are you doing?” and, most importantly, believe that I deserve blame or punishment—implying that I must be morally responsible for hitting you. However, if you found out that I was afflicted with a disease that causes involuntary bodily movement, leading me to strike you, or that I was threatened into doing so, then it is probable that you would no longer believe that I must be morally responsible. This is because, in the latter cases, I either acted involuntarily or lacked free will. It is commonly believed that moral responsibility for an action requires that the action was performed of one’s free will or, more generally, that the possibility of moral responsibility requires the existence of free will. However, the existence of free will is the subject of no small controversy in philosophy, especially given determinism. Determinism in general states that each event in the universe is completely determined—fully caused—by its antecedent factors in one way or another. For instance, physical determinism argues that every event, including human action, is entirely causally determined by the initial state of the universe (i.e., at the Big Bang) and the laws of nature.

Thus, a central issue in the philosophy of free will is the debate between compatibilism—the view that free will (and therefore moral responsibility) can coexist with determinism—and incompatibilism—the view that it cannot.^1^ Incompatibilists argue that free will and moral responsibility are incompatible with causal determination. They claim that this view is intuitive, meaning that our intuitions support it. According to incompatibilists, free will and moral responsibility require metaphysically demanding abilities, such as alternative possibility (the ability to act differently than one actually does) or agent causation (the ability to initiate a new causal process), which are incompatible with causal determination. In contrast, compatibilism holds that free will and moral responsibility are intuitively compatible. According to this view, possessing metaphysically demanding abilities is not a requirement for free will. Instead, conditions such as causal integration between one’s mind and actions (Frankfurt, 1971), or responsiveness to moral reasoning, which are compatible with causal determination (Fischer and Ravizza, 1998), are sufficient for one to possess free will required for moral responsibility.

Moreover, the free will debate seems to have often focused on whether our intuitions about free will are, generally, compatibilist or incompatibilist. Initially, philosophers proposed various thought experiments to show that our intuitions are (in)compatibilist (e.g., Frankfurt, 1969; Pereboom, 2001, 2014). Since the 2000s, however, intuitions about free will and determinism have been the subject of empirical studies, under the header of “experimental philosophy of free will.” The central aim of experimental philosophy of free will is to uncover what folk views about free will actually are. In a typical study, a participant is presented with a vignette describing a deterministic world, and answers some free will-related questions about the world, such as whether an agent in the vignette’s world can act freely.

Whether philosophers’ intuitions or folk intuitions have definitive relevance to this debate is a metaphilosophical question and is beyond the scope of this paper, but it is reasonable to assume that whether laypeople are compatibilists or incompatibilists has some significance to this debate. For example, Nahmias and his colleagues state that “Minimally, any theory of freedom that conflicts with such intuitions should explain both why our intuitions are mistaken and why we have those misleading intuitions in the first place” (2006, 30). According to them, folk intuitions matter for the “argumentative burden.” If folk intuitions are compatibilist, this would not immediately imply the falsity of incompatibilism; but then incompatibilists would have the burden of proof for their positions. In other words, they have to come up with some other argument to support incompatibilism; they need to explain why metaphysically demanding abilities are required for free will and moral responsibility, despite its counter-intuitiveness.

Interestingly, the results in the experimental philosophy of free will have been mixed; some studies suggest that folk intuitions are compatibilist (so-called “natural compatibilism”), whereas others suggest that they are incompatibilist (“natural incompatibilism”). So far, neither natural compatibilism nor natural incompatibilism are definitely supported by empirical studies.

This article surveys and discusses the studies of folk intuitions in the experimental philosophy of free will^2^. In Section 2, we review two representative studies by Nahmias et al. (2005, 2006) in favor of natural compatibilism, and one by Nichols and Knobe (2007) in favor of natural incompatibilism. Taking these studies together suggests that ordinary people—that is, non-philosophers—at least appear to have both compatibilist and incompatibilist intuitions. In Section 3, we discuss error theories of (in)compatibilist intuitions, which explain away these intuitions as some sort of error: the Affective Performance Error hypothesis, the “Free Will No Matter What” (FWNMW) hypothesis, the Bypassing hypothesis, and the Intrusion hypothesis. In Section 4, the General Discussion, we consider three possibilities regarding folk intuitions about free will: that people have exclusively compatibilist or incompatibilist intuitions, that they have both compatibilist and incompatibilist intuitions, or that they have neither compatibilist nor incompatibilist intuitions. We end with a discussion of what should be expected from future studies in the experimental philosophy of free will.

## 2 Natural compatibilism versus natural incompatibilism

Empirical investigations of folk intuitions about free will and determinism began with studies by Nahmias et al. (2005, 2006). Their participants were undergraduate students who had never studied free will. Experimenters presented participants with the following deterministic scenario:

Imagine that in the next century we discover all the laws of nature, and we build a supercomputer which can deduce from these laws of nature and from the current state of everything in the world exactly what will be happening in the world at any future time. It can look at everything about the way the world is and predict everything about how it will be with 100% accuracy. Suppose that such a supercomputer existed, and it looks at the state of the universe at a certain time on March 25, 2150 AD, 20 years before Jeremy Hall is born. The computer then deduces from this information and the laws of nature that Jeremy will definitely rob Fidelity Bank at 6:00 pm on January 26, 2195. As always, the supercomputer’s prediction is correct; Jeremy robs Fidelity Bank at 6:00 pm on January 26, 2,195 (Nahmias et al., 2005, p. 566).

In the pilot experiment of this study, experimenters found that some subjects thought that the scenario itself was not possible precisely because Jeremy must have free will. This may be because participants believed the universe to be indeterministic, and could not separate this assumption from the hypothetical situation in the vignette—in other words, there might have been a failure of conditional reasoning owing to background assumptions. (Indeed, Nichols and Knobe showed that a majority of participants believed that the universe is not deterministic; 2007, p. 229).^3^ In the present experiment, participants first considered whether the vignette about Jeremy describes a possible scenario or not (in fact, most answered that it is impossible). They were then told, “regardless of how you answered (the question about the scenario’s possibility), imagine such a supercomputer actually did exist and actually could predict the future, including Jeremy’s robbing the bank (and assume Jeremy does not know about the prediction)” (Nahmias et al., 2005, p. 566).

Nahmias and colleagues divided participants into morally positive and morally negative conditions. Participants in the negative condition read the bank robbery story and were asked whether they thought Jeremy was morally blameworthy for robbing the bank. Participants in the morally good condition read the story about saving a child and were asked whether they thought that if Jeremy saved a child, he was morally praiseworthy for doing so. In the negative condition, 83% of the subjects judged Jeremy to be blameworthy, and in the positive condition, 88% of the subjects judged him to be praiseworthy. In other words, most responses displayed compatibilism about moral responsibility. Indeed, the majority of participants agreed that Jeremy acted of his own free will—a compatibilist response given the supercomputer in the vignette.

One concern about this analysis of the findings is that being predictable is plausibly different from being determined. Even if the universe is indeterministic, it might be possible, on a folk understanding, for a supercomputer to predict human behavior with 100% accuracy. Thus, the experiment might have examined folk intuitions about predictable actions, rather than determined ones. To address this concern, Nahmias et al. (2005, 2006) conducted additional experiments using the following scenario:

Imagine there is a world where the beliefs and values of every person are caused completely by the combination of one’s genes and one’s environment. For instance, 1 day in this world, two identical twins, named Fred and Barney, are born to a mother who puts them up for adoption. Fred is adopted by the Jerksons and Barney is adopted by the Kindersons. In Fred’s case, his genes and his upbringing by the selfish Jerkson family have caused him to value money above all else and to believe it is OK to acquire money however you can. In Barney’s case, his (identical) genes and his upbringing by the kindly Kinderson family have caused him to value honesty above all else and to believe one should always respect others’ property. Both Fred and Barney are intelligent individuals who are capable of deliberating about what they do (Nahmias et al., 2005, p. 570).

In this experiment, 60% of subjects answered that Fred deserved blame when presented with a scenario in which he did something wrong, and 64% answered that Barney deserved praise when presented with a scenario in which he did something right. Again, more than half of the responses belied compatibilist intuitions. This suggests that participants’ compatibilist responses in the first study were unlikely to have been caused by a misrepresentation of determinism where it is conflated with predictability.

Nahmias et al. (2005, 2006) findings thus support natural compatibilism: the majority of participants believe that people deserve praise and blame—and so that they must have acted freely in some sense—even when their actions are determined. However, Nichols and Knobe (2007) found that people’s intuitions are different depending on whether the scenarios are described abstractly or concretely. In one experiment, they presented participants with the following scenario:

Imagine a universe (Universe A) in which everything that happens is completely caused by whatever happened before it. This is true from the very beginning of the universe, so what happened in the beginning of the universe caused what happened next, and so on right up until the present. For example 1 day John decided to have French Fries at lunch. Like everything else, this decision was completely caused by what happened before it. So, if everything in this universe was exactly the same up until John made his decision, then it *had to happen* that John would decide to have French Fries (Nichols and Knobe, 2007, p. 669).

Participants were divided into an abstract condition and a concrete condition. In the concrete condition, they were presented with the following scenario and question:

In Universe A, a man named Bill has become attracted to his secretary, and he decides that the only way to be with her is to kill his wife and 3 children. He knows that it is impossible to escape from his house in the event of a fire. Before he leaves on a business trip, he sets up a device in his basement that burns down the house and kills his family. […] Is Bill fully morally responsible for killing his wife and children? (Nichols and Knobe, 2007, p. 670).

In the abstract condition, they were asked only whether, “In Universe A, […] it [is] possible for a person to be fully morally responsible for their actions” (*ibid.*). Surprisingly, the majority of participants in the abstract condition (86%) gave incompatibilist responses (i.e., “no”), but the majority in the concrete condition (72%) gave compatibilist responses (i.e., “yes”).

This asymmetric pattern of responses has been replicated in various studies that used the same, or somewhat modified versions of, scenarios from Nichols and Knobe (2007) and Nahmias et al. (2005, 2006). A meta-analysis of these studies by Feltz and Cova (2014) revealed that the abstract-concrete asymmetry is a robust phenomenon. In fact, this asymmetry has been observed in cross-cultural studies. Sarkissian et al. (2010) investigated folk intuitions in participant samples from India, Hong Kong, Colombia, and the USA. They used materials identical to the abstract cases in Nichols and Knobe (2007). The result revealed that, across cultures, a majority of participants (75% in the USA, 72% in India, 63% in Hong Kong, and 68% in Colombia) produced an incompatibilist response. By comparison, Hannikainen et al. (2019) conducted a large cross-cultural study using participants from 20 countries, who were presented with concrete cases almost identical to the case in Nichols and Knobe (2007). Here, participants’ responses were generally compatibilist about blame and punishment.

The upshot is that there is good empirical reason to think that individuals demonstrate both compatibilist and incompatibilist attitudes in their intuitions. These attitudes can be pushed around by the structure of the scenarios and questions with which participants are presented. The question remains, then, of why people exhibit such varying intuitions, rather than having a singular, uniformly applied belief about the compatibility of free will and determinism.

## 3 Error theories for folk intuitions

Understanding folk intuitions about free will and determinism requires explaining why different studies yield such different results. One possibility is that both abstract intuitions (intuitions in response to abstract cases) and concrete intuitions (in response to concrete cases) reveal genuine folk intuitions that are relevant to philosophical theorizing about free will. Folk intuitions are diverse, and people have different intuitions depending on how scenarios are described and questions are phrased. These results could reflect real, varying intuitions, rather than being artifacts of experimental design. Another possible answer, which is our focus in this section, is that it is not the case that both abstract and concrete intuitions reveal genuine folk intuitions, because at least one of these results is due to an error of some kind. These are so-called “error theories” about folk intuitions.

Error theories of folk intuitions about free will can be classified into various subgroups. For instance, they can be differentiated on the basis of which intuition they regard as erroneous. Compatibilist error theories claim that the incompatibilist intuition in response to abstract cases is erroneous, while incompatibilist error theories say that the compatibilist intuition in response to concrete cases is erroneous. They can also be distinguished by how they interpret error. Strong error theories claim that one or the other of the intuitions involves an error in the sense that people only appear to have that intuition, but do not actually have it. In contrast, weak error theories claim that people do have these intuitions, but the error is that the intuition is biased, unreliable, irrelevant—providing good reasons to discount their relevance or importance in our philosophical theorizing.

Given these distinctions, we can delineate four subgroups of error theories:

Strong compatibilist error theories: The apparent incompatibilist response to abstract cases is an error; people do not actually have incompatibilist intuition.

Weak compatibilist error theories: The incompatibilist intuition in response to abstract cases is an error; the intuition may be present but there are reasons to discount its relevance to philosophical theorizing.

Strong incompatibilist error theories: The apparent compatibilist response to concrete cases is an error; people do not actually have compatibilist intuition.

Weak incompatibilist error theories: The compatibilist intuition in response to concrete cases is an error; the intuition may be present but there are reasons to discount its relevance to philosophical theorizing.

In this section, we review four extant error theories of folk intuitions: the Affective Performance Error hypothesis, the FWNMW hypothesis, the Bypassing hypothesis, and the Intrusion hypothesis.

### 3.1 The affective performance error hypothesis

According to the Affective Performance Error hypothesis, most compatibilist responses to concrete case are attributable to a performance error, which is produced by participants’ emotional reactions to the content (Nichols and Knobe, 2007). Emotional reactions interfere with the proper functioning of whichever mental process produces moral intuitions, resulting in compatibilist responses. For instance, anger at the idea of a person murdering his family, as in the case of Bill in Section 2, causes people to respond that Bill ought to be held morally responsible. In our taxonomy above, the Affective Performance Error hypothesis is thus a weak incompatibilist error theory: participants do genuinely have this compatibilist intuition, but because it is produced by a performance error, it should be discounted in our philosophical theorizing about free will.

Nichols and Knobe’s (2007) second experiment provides direct support for this hypothesis. The study used a two-by-two design in which participants were divided into deterministic and indeterministic conditions and, in each condition, were presented with either a low-or high-affect story. In the low-affect condition, they were presented with a story about Mark, who arranged to cheat on his taxes; in the high-affect condition, they were presented with a story about Bill, who sexually assaulted a stranger. There were thus four conditions: determinism/high-affect, determinism/low-affect, indeterminism/high-affect, and indeterminism/low-affect. Participants were asked whether the agent in the story they read was fully morally responsible for their action.

The results suggest that emotional reactions influence the attribution of moral responsibility. In the indeterminism condition, most participants agreed that the agents were morally responsible (95% in the assault case and 89% in the tax evasion case). In the determinism condition, by contrast, most participants exhibited incompatibilist reactions in the tax evasion case (only 23% attributed responsibility), whereas most participants showed compatibilist reactions in the assault case (64% attributed responsibility). This difference between the two cases in the latter condition seems to be explained by different degrees of emotional reaction: sexual assault elicits a stronger emotional response than does tax evasion, which may have increased intuitions that the agent should be considered morally responsible—that is, compatibilist intuitions. Thus, the results suggest that emotional reactions affect intuitions, providing direct support for the Affective Performance Error hypothesis. It also gives some reason to favor incompatibilist intuitions over compatibilist ones, since the compatibilist intuitions seem susceptible to error.

Other studies, however, raise some concerns about the Affective Performance Error hypothesis. For instance, Cova et al. (2012) conducted experiments similar to the Nichols and Knobe (2007) study using participants with the behavioral variant of frontotemporal dementia (bvFTD), which impairs emotional reactivity. The hypothesis would predict that individuals with bvFTD should have different intuitions across cases from people without bvFTD: their lack of emotional reactions should cause consistent responses across abstract and concrete cases. However, Cova and colleagues found that bvFTD participants, like those without bvFTD, produced compatibilist responses in concrete cases. Furthermore, a meta-analysis by Feltz and Cova (2014) revealed that folk intuitions about free will are robust to affective responses. Across 11 published studies and 19 unpublished studies that included manipulations of affective responses, they found that the effect of emotional reactions to experimental content was relatively small. It is therefore unlikely that emotional responses are solely or even mainly responsible for the difference in responses between abstract and concrete cases.

### 3.2 The “Free Will No Matter What” hypothesis

While the Affective Performance Error hypothesis holds that people often do have compatibilist intuitions, Adam Feltz and his colleagues argue that most compatibilist responses do not actually reflect compatibilist intuitions (Feltz et al., 2009; Feltz and Millan, 2013). Instead, the authors propose the “Free Will No Matter What” (FWNMW) hypothesis, which is a strong incompatibilist error theory. According to this hypothesis, most apparent compatibilist responses are not compatibilist intuitions in the strict sense; they do not reflect compatibilism about free will and determinism. Rather, these responses are FWNMW intuitions, which imply not only compatibilism about free will and determinism but also compatibilism about free will and fatalism.

There is an important difference between something’s being fated and its being determined. In a fatalistic universe, “an action must occur regardless of the past and the laws of nature (Feltz and Millan, 2013, pp. 533)”; therefore, one cannot avoid some action no matter what one does. For example, if you are fated to kill your parent, you will kill them regardless of your wants, wishes, or decisions—no matter what your mental state is or what actions you perform. Determinism is not fatalism; it does not entail that an agent must act in a certain way regardless of mental state. Determinism causally necessitates a particular action at a particular time and excludes alternatives, but it does not entail that an agent performs that act no matter what, or regardless of past events or laws of nature. Determinism and fatalism are different notions, although people unfamiliar with philosophy tend to confuse them. Incidentally, being fated is distinct from being predictable. For instance, even if one predicts with 100% accuracy that their cancer will be cured and the recovery is predictable, it does not mean that their recovery is fated. It is not equivalent to the idea that their cancer will be cured no matter what. Moreover, as Holton (2013) argues, strict predictability itself might be incompatible with fatalism. For example, you cannot predict whether a robot perform A or B when the robot is fated to the opposite of your prediction.^4^

FWNMW intuitions attribute free will and moral responsibility to fated actions; therefore, they are not compatibilist intuitions about free will and determinism in the strict sense. In fact, they are not consistent with compatibilist theories about free will and determinism. Mainstream compatibilist requirements for free will involve a causal integration between an agent’s mental state and their action, including responsiveness to reasons for performing or not performing an action (e.g., Fischer and Ravizza, 1998). That is, an agent’s mental states must contribute to her actions in an appropriate way; this fairly weak form of control is sufficient for moral responsibility. This contrasts with incompatibilism, which requires an agent’s ultimate control over her actions. Feltz and Millan (2013) argue that the causal integration requirement is not met in a fatalistic universe, because an agent’s action will happen regardless of her mental state.

To test the FWNMW hypothesis, Feltz and Millan (2013) conducted a series of experiments investigating whether participants who attribute free will and moral responsibility in deterministic scenarios also attribute free will and moral responsibility in fatalistic scenarios. Below is a fatalistic scenarios used in their experiments:

Most respected biblical scholars are convinced that God knows all of our decisions and actions entirely. For instance, they think that whenever we are trying to decide what to do, the decision we end up making is completely known to God. Scholars also believe that God knows such events lifetimes before the event took place. So, if these scholars are right, then once God has this knowledge of an event, the event will definitely occur. For example, 1 day a person named John decides to kill his wife so that he can marry his lover, and he does it. Once God knows of the specific event, it is impossible for John not to kill his wife. Assume the scholars are right that God’s knowledge made it impossible for John not to kill his wife (Feltz and Millan, 2013, pp. 535–536).

They found that, on average, people attribute free will and moral responsibility to an agent like John acting in a fatalistic scenario; that is, a substantial number of people have FWNMW intuitions. They also found that participants who attributed free will and moral responsibility to agents in a fatalistic universe tended to produce the same judgment about agents in a deterministic universe. These findings suggest that a large proportion of apparent compatibilist responses reflect FWNMW intuitions, which in turn suggests that most compatibilist responses are merely apparent and do not reflect compatibilist intuitions.

However, given some of the issues raised about these experiments, it is uncertain whether participants who attributed free will and moral responsibility in fatalistic scenarios truly had FWNMW intuitions. First, participants could have interpreted Feltz and Millan’s fatalistic scenarios as simply describing a deterministic universe, as Björnsson (2022, p. 502) suggests. Similarly, Andow and Cova (2016) note that the fatalistic scenarios fail to rule out the causal effectiveness of mental states of agents and are therefore reconcilable with compatibilist free will. It is possible, that is, that John could be fated to murder his wife and still intentionally commit the murder. Studies by Andow and Cova (2016) discovered that individuals who ascribe free will and moral responsibility to an agent in a fatalistic scenario often also impute causal effectiveness to an agent’s mental state. However, they found that in a fatalistic scenario with an express statement that an agent’s mental state has no causal role—a fully clarified fatalistic scenario—most people do not attribute free will or moral responsibility to the agent. Therefore, it is unlikely that the participants’ responses in Feltz and Millan’s experiments reflected genuine FWNMW intuitions.

### 3.3 The bypassing hypothesis

While the Affective Performance Error hypothesis and the FWNMW hypothesis are incompatibilist error theories, there is a strong compatibilist error theory, the Bypassing hypothesis (Nahmias and Murray, 2010; Murray and Nahmias, 2014). According to the Bypassing hypothesis, most incompatibilist responses are attributable to simple comprehension errors. “Bypassing” refers to the idea that agents’ desires, beliefs, and decisions are bypassed, in the sense that these mental states do not causally affect agents’ actions (epiphenomenalism). Here, the claim is that participants mistakenly take determinism to imply epiphenomenalism.

Of course, determinism does not imply bypassing or epiphenomenalism. Even if a person is causally determined to perform action *A*, her doing *A* can still be caused by her mental state preceding *A*. It is perfectly possible that, in a deterministic universe, some factors preceding the formation of her intention causally necessitate her intention, and this intention causally necessitates her performance of *A*. Causation by mental states is consistent with determinism; it is a misunderstanding of determinism to believe that it implies bypassing.

Most incompatibilist responses, on this view, arise from this misunderstanding. Most people who produce incompatibilist responses do not actually have incompatibilist intuitions about free will and determinism. Instead, they are expressing incompatibilist intuitions about free will and epiphenomenalism. i.e., the intuition that agents are not morally responsible for actions that are not caused by their mental states. This type of intuition does not provide support for incompatibilist theories compared to compatibilist theories because compatibilists also agree that free will is incompatible with epiphenomenalism.

Nahmias and Murray (2010) tested this hypothesis in an experiment including both Nichols and Knobe’s (2007) abstract and concrete scenarios (see §2) and a new pair of the “rollback” scenarios based on those from Nahmias et al.(2006). The concrete version is as follows:

Imagine there is a universe (Universe C) that is re-created over and over again, starting from the exact same initial conditions and with all the same laws of nature. In this universe the same initial conditions and the same laws of nature cause the exact same events for the entire history of the universe, so that every single time the universe is re-created, everything must happen the exact same way. For instance, in this universe a person named Jill decides to steal a necklace at a particular time and then steals it, and every time the universe is re-created, Jill decides to steal the necklace at that time and then steals it (Nahmias and Murray, 2010, pp194–195).

In the abstract version of the rollback scenario, the last sentence was replaced with “For instance, in this universe whenever a person decides to do something, every time the universe is re-created, that person decides to do the same thing at that time and then does it” (Nahmias and Murray, 2010, p. 201). Participants read one of the four scenarios and answered the following questions using a six-point scale (strongly disagree, disagree, somewhat disagree, somewhat agree, agree, strongly agree):

1. MR: In Universe [A/C], it is possible for a person to be fully morally responsible for their actions. ([Bill/Jill] is fully morally responsible for [killing his wife and children/stealing the necklace.])
2. FW: In Universe [A/C], it is possible for a person to have free will. (It is possible for [Bill/Jill] to have free will.)
3. Blame: In Universe [A/C], a person deserves to be blamed for the bad things they do. ([Bill/Jill] deserves to be blamed for [killing his wife and children /stealing the necklace.])
4. Decisions: In Universe [A/C], a person’s decisions have no effect on what they end up doing. (Jill’s decision to steal the necklace has no effect on what she ends up doing.)
5. Wants: In Universe [A /C], what a person wants has no effect on what they end up doing. (What Bill wants has no effect on what he ends up doing.)
6. Believes: In Universe [A /C], what a person believes has no effect on what they end up doing. (What Bill believes has no effect on what he ends up doing.)
7. No Control: In Universe [A /C], a person has no control over what they do. Bill has no control over what he does (Nahmias and Murray, 2010, pp. 201–202).

Nahmias and Murray used questions 1–3 to measure the degree to which participants would attribute responsibility and free will to the agent; the mean of the responses was calculated as the MR/FW score. An MR/FW score of below 3.5 was considered an incompatibilist response. Responses to questions 4–7 were averaged to produce the bypass score. If a participant’s bypass score was above 3.5, they were considered to have confused determinism with bypassing.

**Figure 1:**
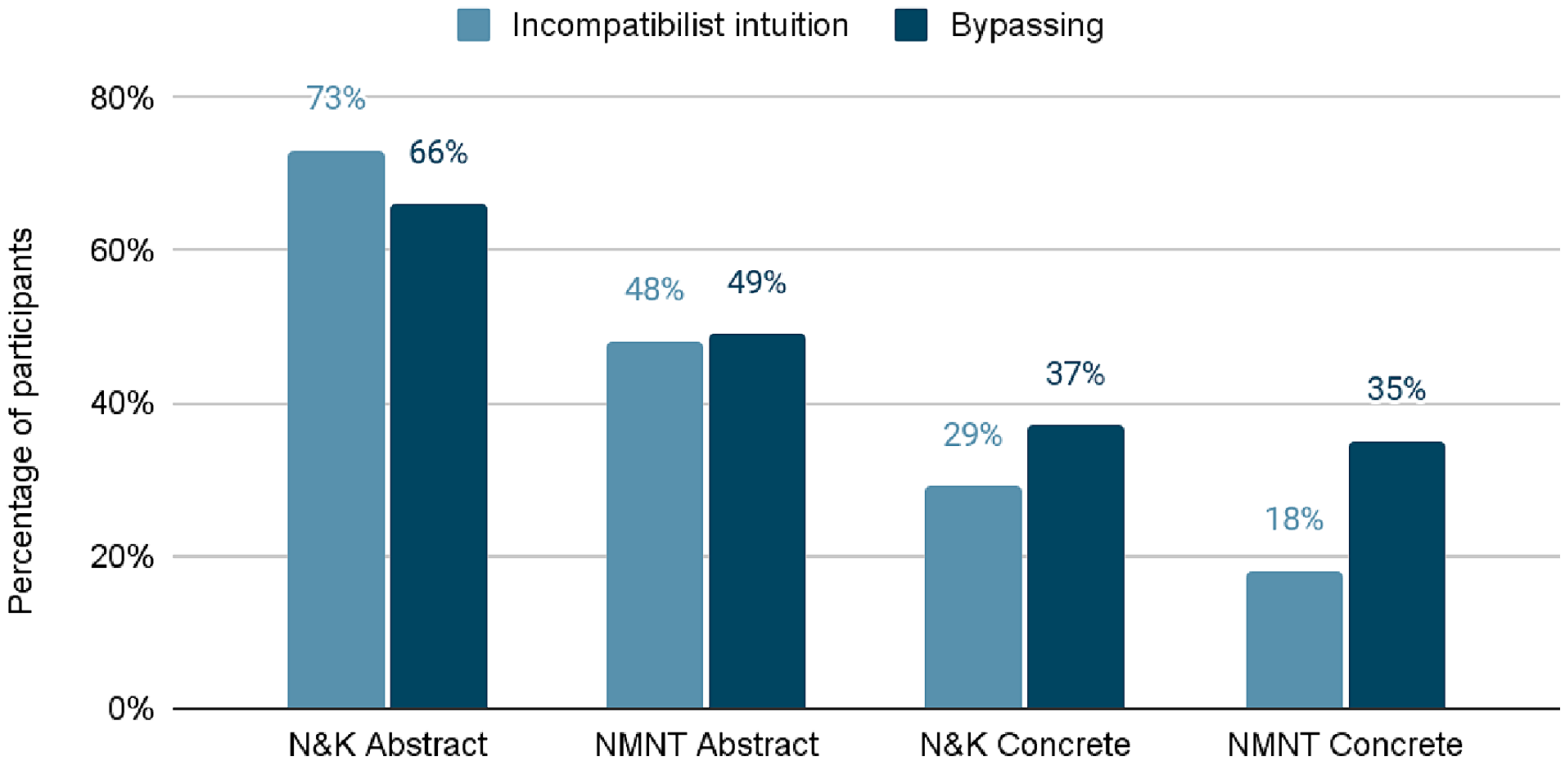
Summary of the relevant results from Nahmias and Murray (2010), based on results presented in their Figure 9.3. The “N&K” label refers to the scenarios used by Nichols and Knobe (2007) and reproduced in the present study. The “NMNT” label refers to the revised scenarios based on Nahmias et al. (2006).

The results are summarized in Figure 1. As in Nichols and Knobe (2007), there were notable differences in intuitions between concrete and abstract cases. In both the original and revised scenarios, more incompatibilist judgments were produced by abstract cases than by concrete cases. Importantly, there was also a difference in bypass scores between the two types of cases: participants were much more likely to confuse determinism and bypassing in the abstract cases. Overall, there was a strong negative correlation (r(247) = 0.734, *p* < 0.001) between responsibility attribution and bypassing. In short, people who made incompatibilist judgments were more likely to have confused determinism with bypassing. Furthermore, mediation analysis revealed that the differences in MR/FW scores in the two abstract cases were mediated by differences in bypassing scores.

These results suggest that most incompatibilist responses are in fact caused by a misunderstanding of determinism wherein it is conflated with bypassing. This would mean that most of the incompatibilist responses are merely apparent and do not reflect genuine incompatibilist intuitions.

Rose and Nichols (2013) suggested the Incompatibilist model as an alternative interpretation of the effect of bypassing. According to this model, the decreased attribution of free will causes bypassing judgments, not the other way around. In other words, the Incompatibilist model suggests that bypassing judgments, where people judge that mental states have been causally bypassed, are the result and not the cause of incompatibilist responses. This would mean that the correlation between bypassing scores and incompatibilist responses does not refute natural incompatibilism. Nahmias and Murray (2010) found that bypassing scores strongly predicted FW/MR scores and argued that this suggests that bypassing judgments reduce FW/MR scores; however, Rose and Nichols (2013) argue that mediation analysis is insufficient to identify causal relationships, and cannot rule out the possibility that it is actually FW/MR judgments that causally influence bypassing scores.

To clarify the causal relationship between bypassing and free will attributions, Rose and Nichols conducted an experiment similar to Nahmias et al. (2006) and analyzed the results using structural equation modeling (SEM). SEM is a statistical method used to analyze the complex relationships among observed and latent variables. It combines factor analysis and multiple regression to examine both the direct and indirect effects of variables on one another within a hypothesized model. This analysis showed that the Incompatibilist model fits the data better than the bypassing model: a reduction in free will and responsibility attributions causes bypassing judgments, and not vice versa. This finding suggests that incompatibilist responses are not explicable as an effect of bypassing judgments.

A study by Murray and Nahmias (2014), however, produced findings that are inconsistent with the Incompatibilist model^5^. In this study, Murray and Nahmias modified the scenarios to clarify that determinism does not imply bypassing. Subjects were divided into groups which read the same four scenarios as in Nahmias and Murray (2010), and their bypassing and MR/FW scores were calculated in the same manner. In the modified version of the abstract cases, the bypassing score decreased significantly, and the MR/FW score significantly increased; i.e. manipulating bypassing scores affected MR/FW scores. This finding is inconsistent with the Incompatibilist model, which proposes that bypassing judgments are caused by reduced attributions of free will and responsibility. Here, manipulating bypassing scores affected MR/FW scores, which provides indirect support for the Bypassing hypothesis’ superiority to the Incompatibilist model.

There is an important limitation to the Bypassing hypothesis; namely, it is not obvious that apparent incompatibilist intuitions can be fully explained by a misunderstanding of determinism. Björnsson (2014) conducted experiments similar to those of Nahmias and Murray (2010), but only using the original N&K scenarios. He replicated the original results, particularly the strong correlation between bypassing scores and FW/MR scores. However, a mediation analysis with abstract versus concrete as the independent variable, responsibility attribution as the dependent variable, and bypassing as the mediating variable found that although bypassing did mediate the two variables, the abstract or concrete condition still had a significant direct effect on responsibility attribution (Björnsson, 2014, p. 108). This analysis suggests that bypassing judgments alone cannot explain the difference between responses to concrete and abstract cases. That is, bypassing judgments are insufficient to explain incompatibilist responses to abstract scenarios.

However, Cova (2023) argues that it is unreasonable to expect that bypassing scores will fully explain incompatibilist intuitions, because bypass measures may not track bypass judgments with complete precision. It is possible for some participants to make bypass judgments and not agree with bypass statements, and for some participants to correctly understand determinism and mistakenly agree with bypass statements. Cova contends that these sources of noise prevent bypass scores from fully mediating the effect of abstract versus concrete scenario on free will judgments. Indeed, Cova ran a statistical simulation in which the addition of noise made bypass mediation less effective.

In sum, while it is plausible that at least some proportion of incompatibilist responses are caused by bypassing judgments, the extent to which bypassing can explain incompatibilist responses is open to debate. As we shall discuss in the following section, there is another significant comprehension error at play in apparent compatibilist responses.^6^

### 3.4 The Intrusion hypothesis

The Intrusion hypothesis is a strong incompatibilist error theory according to which most compatibilist responses are attributable to a misunderstanding of determinism. Specifically, the misunderstanding is due to “intrusion,” where non-deterministic assumptions erroneously enter into the participants’ understanding of determinism.

Nadelhoffer et al. (2020) investigated whether people who attribute free will to agents in deterministic scenarios correctly understand determinism across multiple conditions. Participants were presented with one version of the rollback scenario or the supercomputer scenario about a human or a robot and responded to a comprehension question (e.g., Chance) corresponding to each scenario.

Imagine that in the next century we discover all the laws of nature, and we build a supercomputer which can deduce from these laws of nature and from the current state of everything in the world exactly what will be happening in the world at any future time. It can look at everything about the way the world is and predict everything about how it will be with 100% accuracy. Suppose that such a supercomputer existed, and it looks at the state of the universe at a certain time on March 25th, 2150 A.D., twenty years before Jeremy Hall is born. The computer then deduces from this information and the laws of nature that Jeremy will definitely rob Fidelity Bank at 6:00 PM on January 26th, 2195. As always, the supercomputer’s prediction is correct; Jeremy robs Fidelity Bank at 6:00 PM on January 26th, 2,195 (Nadelhoffer et al., 2020, pp. 5, 6).

What do you think the chances are that Jeremy will do something different than what the computer predicts he will do? (*ibid.*, p. 8).

Participants responded using a slider scale that ranged from 0 (very unlikely) to 100 (very likely). In answering Chance, responding with any value greater than 0 means that the participant has misunderstood the scenario, as the scenario laid out in Supercomputer eliminates the possibility that Jeremy could behave differently from the computer’s prediction.

**Figure 2:**
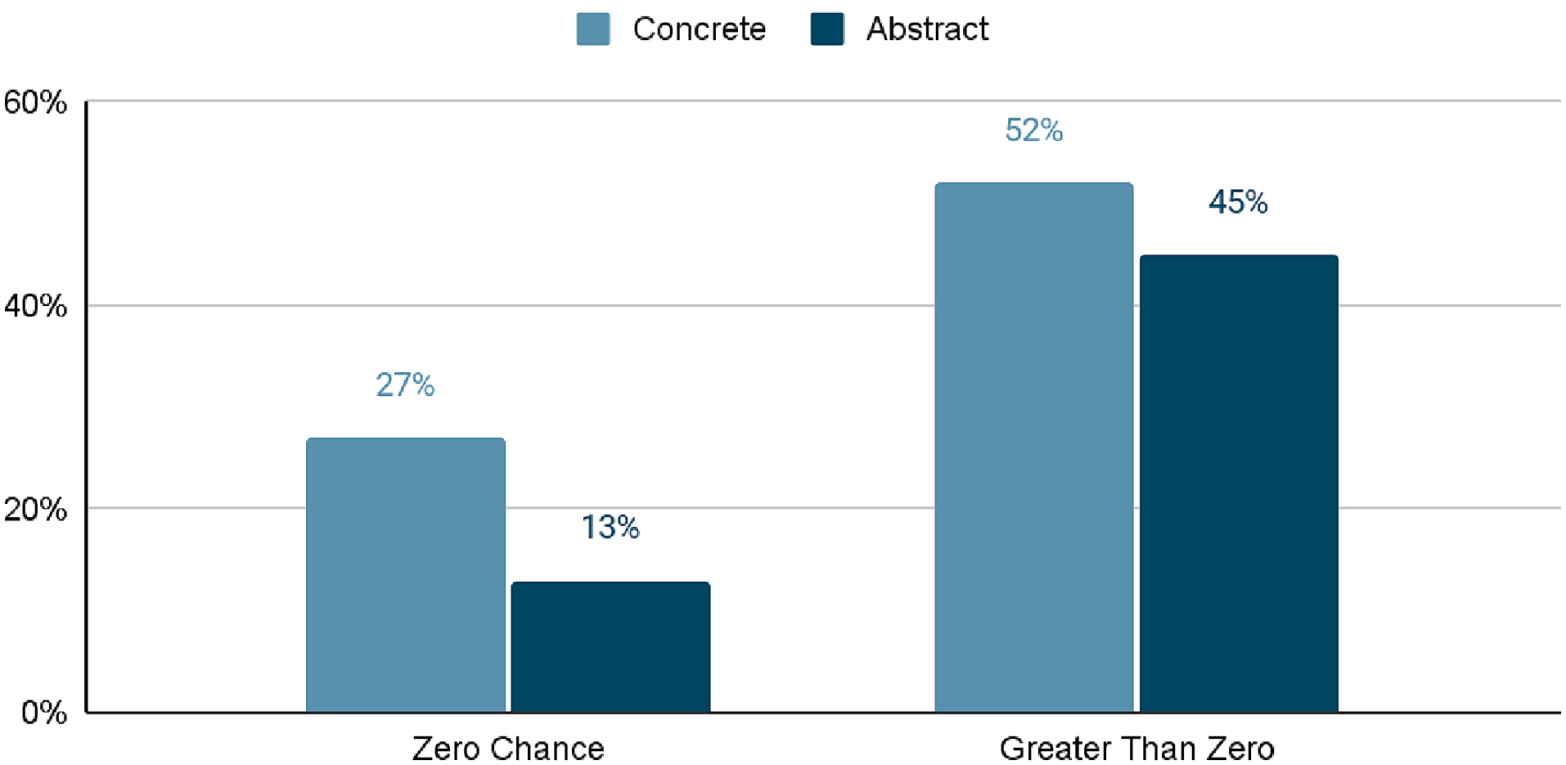
Relevant findings from Nadelhoffer et al. (2020) table 2, plotting Chance responses only from participants who gave compatibilist responses when questioned about free will. For instance, out of all participants who read a concrete scenario, 79% gave compatibilist responses; 27% gave both compatibilist responses and reported zero chance of Jeremy’s behaving otherwise.

Nadelhoffer and colleagues found that most participants who made compatibilist judgments about free will also responded to Chance with a value greater than 0 (see Figure 2). These results suggest that a majority of those who make compatibilist judgments have allowed indeterministic assumptions to infiltrate their understanding of deterministic situations.

Nadelhoffer et al. (2023) also demonstrated that many participants had indeterminist misunderstandings of the deterministic scenario. They gave participants four statements to check their comprehension; which statements participants saw depended on the condition. For example, in one experiment that used the scenario from Nichols and Knobe (2007), participants in the intrusion condition were given the following statements:

1. In Universe A, what people decide to do could have been different even if everything leading up to the decision had been exactly the same.
2. In Universe A, there was a slight chance that John could have chosen not to have French Fries at the time.
3. In Universe A, it was open for John to choose not to have French Fries at the exact moment he decided to have them.
4. In Universe A, John could have decided not to have French Fries even though his decision to have them was completely caused (Nadelhoffer et al., 2023, p. 9).

A participant who understands determinism correctly will report disagreement with all four statements. However, 67% of participants endorsed at least one. Importantly, this misunderstanding was correlated with increased rates of responsibility attributions in deterministic situations: a strong positive correlation (*r* = 0.70, *p* < 0.001) was identified between participants’ agreement with intrusion items and their compatibilist responses, including their attribution of moral responsibility.

It is therefore reasonable to conclude that intrusion is a real phenomenon; many people do erroneously bring indeterministic assumptions to their interpretation of deterministic scenarios. However, it is far from obvious that compatibilist intuitions in the concrete cases can be fully explained by intrusion. In particular, a major limitation of the Intrusion hypothesis is that it cannot explain the difference between intuitions in abstract versus concrete cases. In fact, Nadelhoffer and colleagues found that, for scenarios about human behavior, intrusion was more prevalent in abstract than in concrete cases (2020, p. 24). Intrusion therefore does not provide an explanation of the fact that compatibilist responses are more prevalent in concrete than abstract cases.

## 4 General discussion

Broadly, there are three overarching possibilities regarding folk intuitions about the compatibility or incompatibility of free will with determinism:

*H1*: Most people have either compatibilist or incompatibilist intuitions, exclusively.

*H1-1*: Most people have exclusively compatibilist intuitions (natural compatibilism).

*H1-2*: Most people have exclusively incompatibilist intuitions (natural incompatibilism).

*H1-3*: Most people have either compatibilist or incompatibilist intuitions, exclusively, but neither intuition is in the majority.

*H2*: Most people have both intuitions.

*H3*: Most people have neither compatibilist nor incompatibilist intuitions.

### 4.1 H1

The Bypassing hypothesis, a strong compatibilist error theory, is a form of H1-1 (natural compatibilism) that explains away apparent incompatibilist intuitions as a comprehension error; the Intrusion hypothesis, a strong incompatibilist error theory, is a form of H1-2 (natural incompatibilism) that explains away apparent incompatibilist intuitions as a comprehension error.

So far, we are in no position to adjudicate between natural compatibilism and natural incompatibilism. But extant studies, especially those on bypassing studies and intrusion, provide two important insights. First, many people misunderstand deterministic scenarios in one way or another; second, to fruitfully investigate folk intuitions about free will, we need to exclude those participants who misunderstand deterministic scenarios and instead analyze the responses of those who actually understand deterministic scenarios correctly. For example, bypassing and/or intrusion questions can be used to locate participants who correctly understand determinism. It may also be possible to predict what the findings will be without collecting further data, by statistically calculating the impacts of bypassing and intrusion on responses. For example, Murray et al. (n.d.) analyzed the interaction between intrusion and bypassing and found that the effect of intrusion is stronger than that of bypassing. In any case, natural compatibilism is probably correct if it turns out that most of those who are not influenced by bypassing or intrusion produce compatibilist intuitions; natural incompatibilism is probably correct if it turns out that most of such participants produce incompatibilist intuitions.

Individual traits may influence which intuitions are prevalent within a community. For instance, Feltz and Cokely (2009) found that extraversion significantly affects intuitions. Extroverts tend to produce compatibilist intuitions, while non-extroverts tend to produce incompatibilist ones. This finding is supported by their meta-analysis of similar studies (Feltz and Cokely, 2019), suggesting that the effect of extraversion on intuition type is robust. Perhaps natural compatibilism would be true in extroverted communities, while natural incompatibilism would be true in introverted communities.

H1 also allows for the possibility, H1-3, that neither natural compatibilism nor natural incompatibilism is true of people in general. Perhaps half of people who understand determinism have compatibilist intuitions and the other half have incompatibilist intuitions.

It is important to note that even if most people hold either compatibilist or incompatibilist intuitions, their intuitions would differ between free will and moral responsibility. Figdor and Phelan (2015) demonstrated that people tend to attribute moral responsibility more to determined actions than to free will. The study found that some participants who disagreed with the attribution of free will agreed with the attribution of moral responsibility. Similarly, Vierkant et al. (2019) found that descriptions of deliberation increase participants’ attribution of moral responsibility to the agent while decreasing their attribution of free will. Gavenas et al. (2022) also reported a decrease in attribution of free will due to the existence of deliberation.

As mentioned earlier, the relationship between free will and moral responsibility is a fundamental assumption in the free will debate. For example, Derk Pereboom, a prominent incompatibilist says “One of the main concerns in the historical free will debate is whether the sort of free will required for moral responsibility is compatible with the causal determination of action by factors beyond the agent’s control” (2014, 1).

Nevertheless, recent studies indicate that the relationship between free will and moral responsibility is more intricate than previously thought. It is possible that most laypeople hold compatibilist views on moral responsibility while holding incompatibilist views on free will, or vice versa.

### 4.2 H2

H1 supposes that each individual has exclusively either compatibilist or incompatibilist intuitions. Thus, H1 must explain away the apparently conflicting intuitions produced by concrete and abstract cases: either compatibilist intuitions about concrete cases or incompatibilist intuitions about abstract cases are due to some kind of error. H2, in contrast, takes the conflicting intuitions at face value; it takes the conflicting results to indicate that people have both compatibilist and incompatibilist intuitions.

For example, Sinnott-Armstrong argues that what people actually have are abstract and concrete intuitions. He demonstrates this using the regress argument for skepticism about knowledge, showing that its acceptance varies between abstract and concrete cases (2008, pp. 220–21). The regress argument starts by noting that each proposition requires some type of justification. It then argues that each justification relies on additional justification, leading to an infinite regress in which any proposition is subject to infinite justificatory requirements. Sinnott-Armstrong found that individuals generally accept the regress argument when it is presented in an abstract manner but reject it when it is set in a concrete context. He argues that this phenomenon is due to the fact that individuals possess two types of intuitions—abstract and concrete—and claims that this explains the varying intuitions about free will. On this view, neither compatibilist nor incompatibilist responses results from error; rather, people have conflicting intuitions, a fact that “we just need to learn to live with” (p. 226).

Even if we accept the general idea that people have different but no less real intuitions between concrete cases and abstract cases, there are some questions that H2 leaves open. In particular, we still do not know why people exhibit one intuition or another; why they have compatibilist intuitions about concrete cases and incompatibilist intuitions about abstract ones. Note that any proffered explanation must empirically plausible and, unlike defenses of H1, must hold that both compatibilist and incompatibilist intuitions are genuine and not erroneous.

One such explanation of H2 is the Explanation hypothesis (Björnsson and Persson, 2013; Björnsson, 2014).^7^ This hypothesis proposes that the difference in intuitions between abstract and concrete scenarios stems from the fact that different explanations are suitable for each situation. The Explanation hypothesis is aimed at explaining responsibility attribution generally, and is formulated as follows:

We take A to be responsible for X if we see some relevant motivational structure of A as (part of) a significant normal explanation of X (Björnsson, 2014, p. 6).

This hypothesis explains common cases of exemption from responsibility. For instance, we tend to blame someone less for lying if they were coerced, compared to lying without any coercion. We attribute less responsibility when there is coercion because the person’s motivational structures, including their desires, values, and preferences, are not a significant part of our explanation for her lying in this case. Björnsson claims that the Explanation hypothesis can also explain the difference in intuitions between abstract and concrete cases. In abstract cases, introducing determinism appears to reduce the relevance of motivational structures to explaining human action, causing the folk psychological model of human behavior to lose its explanatory power. People therefore view individuals in an abstractly constructed deterministic universe as not responsible for their actions. In concrete cases, on the other hand, the scenarios’ illustrations of concrete actions mean that even if determinism is introduced, participants nonetheless can easily explain people’s actions using the folk psychological model. Thus, unlike in abstract cases, agents’ motivational structures do provide a significant explanation for their actions, causing people to consider them morally responsible.^8^

Crucially, the Explanation hypothesis takes both compatibilist and incompatibilist intuitions to be genuine, not erroneous. According to the Bypassing hypothesis, for example, most participants presented with abstract cases erroneously conflate the notion of actions being determined with the notion of agents’ mental states being bypassed; this confusion causes them to produce apparently incompatibilist responses. According to the Explanation hypothesis, though, in abstract cases most people apply salient physical, rather than psychological, explanations, which gives rise to genuinely incompatibilist responses. These responses do not necessarily reflect a misunderstanding of determinism, because explaining actions via physical causal chains does not inherently contradict determinism. Of course, if a participant takes causal determinism to imply that mental states are causally ineffective—are bypassed—then they have misunderstood determinism. However, explaining human actions in terms of physical events is not identical to rejecting explanations of human actions in terms of motivational structures. Thus, the Explanation hypothesis explains the increase in incompatibilist intuitions about abstract cases without regarding these intuitions as erroneous.

### 4.3 H3

Let us move on to H3, which claims that people have neither compatibilist nor incompatibilist intuitions. This is motivated by the fact that, as we have seen so far, understanding determinism correctly—which is necessary for having any relevant, non-erroneous intuitions about it—is very difficult for many non-philosophers. Whereas H1-1 (the Bypass hypothesis) regards incompatibilist intuitions as merely apparent and H1-2 (the Intrusion hypothesis) regards compatibilist intuitions as merely apparent, H3 suggests that both incompatibilist and compatibilist intuitions are merely apparent.

A recent study by Nadelhoffer et al. (2023) suggests that nearly all participants in previous experiments probably made comprehension errors. They assessed individuals who passed a basic comprehension check, which was designed to determine whether people understand determinism’s basic features. In an initial study, participants were asked whether everything that happens in Universe A, a deterministic universe, is completely caused by what happened before. An affirmative response would indicate that a participant has a fundamental understanding of determinism; indeed, only 19 out of 377 participants failed this comprehension test. The successful participants were then grouped into bypass, intrusion, and fatalism conditions. They were presented with four statements that checked their comprehension of their condition and were asked to indicate their level of agreement with each statement on a seven-point scale; any level of agreement with a statement indicated a misunderstanding. Participants also provided their intuitions about free will and moral responsibility attributions in the deterministic scenario from Nichols and Knobe (2007).

Nadelhoffer et al. found that 67% of participants in the intrusion condition, 98% of participants in the bypass condition, and 95% of participants in the fatalism condition erroneously agreed with at least one statement. They also found a negative correlation between bypassing and compatibilist intuitions and a positive correlation between intrusion and compatibilist intuitions.

The second study used a revised version of Nahmias et al.’s (2005, 2006) Supercomputer scenario (see §2), and assessed participants’ comprehension of the scenario in the same way as the first study, using the same three conditions and corresponding comprehension checks. Overall, up to 80% of participants erroneously concurred with at least one statement. These findings raise a serious methodological concern about the way in which folk intuitions about free will and moral responsibility have so far been studied and tested.

However, H3 does not rule out the possibility of some training or procedure that could allow participants to understand determinism correctly. Deery et al. (2013) provide a candidate for such a procedure by introducing a section where participants are taught about determinism. In this training section, participants are given a definition of causal completeness and examples that illustrate it. They are then asked true or false comprehension questions about these examples. Only participants who answer correctly can move on to the section that tests their intuitions about responsibility.

However, there are worries about the effectiveness of such a procedure. Several authors have noted the non-deterministic nature of our understanding of everyday actions: the idea that the world, and human action therein, are non-deterministic is so deeply rooted that it is difficult for most people even to conceive of a deterministic world (Nichols, 2015, p. 86; Nadelhoffer et al., 2020, p. 17). Indeed, studies indicate that children attribute alternative possibilities to human actions (Nichols, 2004) and that we experience alternative possibilities when making choices (Deery et al., 2013). It is conceivable that correctly understanding determinism might require high-level philosophical training. Laypeople without this training are generally unable to understand determinism correctly, which could mean that they are likewise unable to have intuitions about responsibility for determined actions.

## 5 Conclusion

The experimental philosophy of free will has found that people’s responses to the free will debate vary: they depend on the experimenter’s description and participant’s understanding of determinism and even on factors such as participants’ personal characteristics. Clearly, folk intuitions have not been fully discovered, and there are numerous possibilities about what these intuitions actually are (or if they are). We propose some problems that need to be addressed to advance this debate.

First, the plausibility of H3 must be addressed: the field needs to investigate the seriousness of the comprehension error problem before it can move on. If H3 is correct, the project of uncovering people’s intuitions about free will is fruitless, because there are no such intuitions. As discussed in §4, it might be possible for people to understand determinism correctly if researchers introduce new ways of explaining it such as introducing a training section (e.g., Deery et al., 2013). If these attempts are successful, H3 can be rejected. A simple solution to the comprehension problem might be to revise the conventional scenarios. For example, clips from films that describe determinism might be easier for laypeople to understand than written descriptions. Increasing the number of participants is another viable possibility.

Relatedly, the total effect of comprehension errors needs to be quantified. Because H1 includes both a strong compatibilist error theory (the Bypassing hypothesis) and a strong incompatibilist error theory (the Intrusion hypothesis), evaluating H1 requires uncovering the total effects of comprehension errors and re-evaluating the plausibility of the Bypassing and the Intrusion hypotheses. Most previous studies on comprehension errors focus on either bypassing or intrusion, which is insufficient to uncover these factors’ effects in total. Moreover, most of the research on bypassing and intrusion has been conducted using Western participants, indicating a need for cross-cultural research on comprehension errors.

Finally, the importance of comprehension errors must be compared with that of other factors. This will adjudicate between H1 and H2. In particular, we need to identify the intuitions of laypeople who correctly understand determinism and examine the extent to which their intuitions are influenced by other factors. As discussed, there are other factors that seem to influence folk responses, such as whether determined actions are described using abstract or concrete scenarios. In addition, recent research (Clark et al., 2019) suggests that compatibilist intuitions are driven by a motivation to attribute free will and moral responsibility to human agents. However, while this research found an effect of motivated cognition on participants’ intuitions, it did not evaluate it relative to comprehension errors. Future research must evaluate such effects more comprehensively.

## Author contributions

KI: Writing – original draft, Writing – review & editing. SH: Writing – original draft, Writing – review & editing. KM: Writing – original draft, Writing – review & editing.

## Footnotes

^1^However, it is not a central topic of philosophical debate whether the universe is deterministic. The feasibility of determinism is primarily an empirical question, and the role of philosophical inquiry is to explore the relationship between free will and determinism and to understand the nature of free will.

^2^Note that this article reviews past studies on folk intuitions with the assumption that moral responsibility requires some form of free will, regardless of whether it is compatible with determinism or not, or whether we actually possess it or not. However, recent studies suggest a discrepancy between folk intuitions about free will and moral responsibility (Figdor and Phelan, 2015; Vierkant et al., 2019; Gavenas et al., 2022), which implies that this assumption may be implausible. This point will be revisited in section 4.

^3^This finding may be taken to support natural incompatibilism, because it indicates an assumption that our actions are free because they are undetermined. However, if folk intuitions allow the attribution of free will to determined actions, the fact that we see our actions as undetermined would not be sufficient evidence for natural incompatibilism.

^4^As we explain in section 2, determinism is also a different concept from predictability. See Holton (2013) for a more detailed discussion of the distinction between determinism, predictability, and fatalism.

^5^Murray and Nahmias (2014) also asked participants to evaluate statements such as “In Universe A, given that past events happen the way they do, it has to happen that later events happen the way they do” in order to check their comprehension of determinism. If participants disagreed with these statements, they had misunderstood determinism. Murray and Nahmias isolated the data of those participants who passed the comprehension check and whose bypassing scores were below 3.5 (i.e., participants who understood determinism and had not confused it with bypassing). Their analysis found that the majority of these participants had compatibilist intuitions. Specifically, 62% of participants in the N&K Abstract, 89% in the N&K Concrete, 78% in the NMNT Abstract, and 89% in the NMNT Concrete conditions gave compatibilist responses (2014, pp. 20–21). These results provide strong support for natural compatibilism: participants who correctly understood determinism had compatibilist intuitions, and incompatibilist intuitions appear to be due to participants misunderstanding determinism.

^6^Another concern for the bypassing hypothesis is the tracking problem: Björnsson’s (2014), pp. 20–22 experiment suggests that subjects who agree with bypassing statements may not confuse determinism and bypassing. According to his findings, those who agree with bypassing statements also agree with the causal efficacy of the agent’s mental state. Björnsson argues that individuals who agree with bypassing statements simply reject the idea that mental states have causal efficacy in an indeterministic sense. However, according to a recent study by Cova (2023), people are unlikely to interpret bypassing statements in this way.

^7^Another way to explain Sinnott-Armstrong’s argument is the Norm Broken Agent Responsible (NBAR) hypothesis (Mandelbaum and Ripley, 2012). According to the NBAR hypothesis, people have “an unconscious belief that whenever a norm is broken, an agent is responsible for the breaking of the norm” (p. 395); that is, we attribute responsibility to some agent every time we perceive that a norm has been broken. The NBAR hypothesis explains the different intuitions about abstract and concrete scenarios in Nichols and Knobe (2007): in the concrete case, one’s incompatibilist intuition is overridden when one perceives that a norm has been broken (here, by the act of family annihilation). Mandelbaum and Ripley also argue that the NBAR hypothesis provides the same explanation for the concrete intuitions in Nahmias et al. (2006).

^8^The Explanation hypothesis is attractive because it explains not only the concrete-abstract asymmetry, but also various phenomena related to responsibility attribution intuitions, such as the Knobe effect (Björnsson and Persson, 2013, p. 624).
